# Using Genetics to Investigate Relationships between Phenotypes: Application to Endometrial Cancer

**DOI:** 10.3390/genes15070939

**Published:** 2024-07-18

**Authors:** Kelsie Bouttle, Nathan Ingold, Tracy A. O’Mara

**Affiliations:** Cancer Research Program, QIMR Berghofer Medical Research Institute, Brisbane, QLD 4006, Australianathan.ingold@qimrberghofer.edu.au (N.I.)

**Keywords:** genome-wide association study, endometrial cancer, cross-phenotype, statistical genetics

## Abstract

Genome-wide association studies (GWAS) have accelerated the exploration of genotype–phenotype associations, facilitating the discovery of replicable genetic markers associated with specific traits or complex diseases. This narrative review explores the statistical methodologies developed using GWAS data to investigate relationships between various phenotypes, focusing on endometrial cancer, the most prevalent gynecological malignancy in developed nations. Advancements in analytical techniques such as genetic correlation, colocalization, cross-trait locus identification, and causal inference analyses have enabled deeper exploration of associations between different phenotypes, enhancing statistical power to uncover novel genetic risk regions. These analyses have unveiled shared genetic associations between endometrial cancer and many phenotypes, enabling identification of novel endometrial cancer risk loci and furthering our understanding of risk factors and biological processes underlying this disease. The current status of research in endometrial cancer is robust; however, this review demonstrates that further opportunities exist in statistical genetics that hold promise for advancing the understanding of endometrial cancer and other complex diseases.

## 1. Introduction

Genome-wide association studies (GWAS) play a pivotal role in advancing the identification of genotype–phenotype associations. GWAS entail a comprehensive examination of common and lower-frequency variants (minor allele frequency > 0.1%) across the entire genome for genetic markers, predominantly single nucleotide polymorphisms (SNPs), demonstrating statistically significant (*p* value < 5 × 10^−8^) associations with specific traits or complex diseases. Identification of genetic risk loci through GWAS allows for the discovery of interventions, identification of high genetic risk groups, and guidance of treatment protocol [1].

Traits or diseases often present with shared clinical and epidemiological risk factors and can be linked through molecular, biological, and population-based data. GWAS have facilitated the exploration of relationships between different diseases and phenotypes through analytical advancements in genetic association, correlation, cross-trait locus identification, and causal inference testing. These methods all have a key role in the analysis of genetic data, offering unique insight into the genetic architecture of complex traits and diseases. Genome-wide genetic correlation measures the overall genetic similarity between two traits by evaluating the average effect of shared genetic variants across the entire genome. While local genetic correlation focuses on individual regions of the genome to identify genetic similarities between traits. Cross-trait GWAS involves the joint analysis of multiple genetically correlated traits to enhance statistical power to uncover pleiotropic loci. Causal inference analysis, such as Mendelian randomization, uses genetic variants as instrumental variables to infer causal relationships between traits. Together, these approaches can provide information on comorbidities for particular diseases, modifiable risk factors and enable cross-phenotype GWAS meta-analyses to identify new genetic loci.

This narrative review will present methods developed using GWAS data to explore relationships between different phenotypes (Table in Section 3) and, where feasible, discuss their application to endometrial cancer, the most commonly diagnosed gynecological cancer in developed countries. This review prioritizes studies incorporating cross-trait genetic analyses pertinent to endometrial cancer. In cases where specific statistical methods had not yet been applied to endometrial cancer, studies on related phenotypes were considered, emphasizing recent publications, large sample sizes, established biological relevance, and peer-reviewed sources.

## 2. Endometrial Cancer

Endometrial cancer has witnessed noteworthy trends in incidence and mortality in recent years. The incidence has surged by over 132% in the past 30 years, with 417,000 new diagnoses worldwide in 2020 [2,3]. While the incidence rates vary globally, there has been a discernible increase in developed nations, likely attributed to factors such as ageing populations and the rising prevalence of obesity [2,4]. There is a well-established link between obesity and endometrial cancer, with excess adiposity leading to increased levels of estrogen, thereby augmenting the risk of developing this malignancy [5]. Although endometrial cancer is typically diagnosed in post-menopausal women at around 60 years of age, up to five percent of cases occur in women under the age of 40 [4].

The leading theoretical pathogenetic pathway for endometrial carcinoma involves prolonged exposure to elevated estrogen levels, whether from exogenous or endogenous sources, which stimulates unopposed endometrial proliferation without adequate opposition by progestin [4,5]. In addition to obesity, other established risk factors include diabetes, polycystic ovary syndrome (PCOS), hypertension, and lifestyle factors like smoking and diet, which all may indirectly influence estrogen levels [6,7]. Direct estrogen-related factors, such as the use of combined oral contraceptive pills for risk reduction or unopposed estrogen replacement therapy for increased risk, further contribute to the disease’s dynamics [8]. Reproductive factors like early menarche, late menopause, and nulliparity also play significant roles [8].

Epidemiological observations and family-based studies have demonstrated that the genetic effect of endometrial cancer is significant, with heritability estimates ranging from 27% to 52% and a two-to-threefold increased risk associated with a family history of endometrial cancer [9,10,11,12]. Rare pathogenic germline variants within mismatch-repair genes (i.e., *MLH1*, *MSH2*, *MSH6*, *PMS2*, and *EPCAM*) indicated an initial genetic predisposition for endometrial cancer in women with Lynch syndrome [13,14]. Despite their rarity in the general population, estimates suggest that these high-risk germline variants contribute to 3% of endometrial cancer cases [15,16].

The genetics of endometrial cancer, particularly elucidated through GWAS, have significantly advanced our understanding of the disease’s etiology, functional mechanisms, and translational implications, demonstrating the effects of common genetic variation (minor allele frequency > 1%) on endometrial cancer risk [17,18]. The largest endometrial cancer GWAS to date, conducted by the Endometrial Cancer Association Consortium (ECAC), used data from nearly 13,000 cases and identified 16 genetic loci associated with endometrial cancer risk [19]. Risk estimates for these common variants individually are expected to only slightly increase the risk associated with endometrial cancer; however, cumulatively they explain about 28% of the familial relative risk [17]. The identified 16 variants are estimated to account for only a quarter of the variance that genetics can explain, implicating a further potential for discovery in unidentified genetic loci.

Increases in sample size and availability of GWAS summary statistics, in addition to larger genetic cohorts, will improve power and facilitate the discovery of genetic risk regions. Patterns of shared genetic influence combined with existing epidemiological observations promise to elucidate functional pathways and contribute to our understanding of the biological underpinning and comorbidity of diseases.

## 3. Genetic Correlation

Genetic correlation is a critical metric to quantify the overall genetic similarity between complex traits irrespective of environmental confounders, which are prevalent and mostly unavoidable in conventional epidemiologic studies. Genetic correlation (denoted as r_g_) ranges from 0 to 1 and describes the average effect of pleiotropy across all causal loci. It is frequently used in the initial identification of associations between traits of interest. Several methods to assess genetic correlation have been developed, summarized in Table 1.

The most commonly used approach for genetic correlation estimation is linkage disequilibrium (LD) score regression, mainly owing to its computational efficiency and use of GWAS summary statistics [24]. LD score regression has been used for an array of diseases and phenotypes, uncovering potentially shared genetic architecture between schizophrenia and a range of psychiatric, metabolite, personality, immune, cardiovascular, substance-related, and anthropometric traits [24,48,49,50,51]. Interestingly, epidemiological studies have previously reported both opposing and direct comorbidity between schizophrenia and several cancer types [52,53]. LD score regression has also estimated a significant positive genetic correlation between schizophrenia and breast cancer, which may partly explain the epidemiological bidirectional association between the two traits and suggests shared biological mechanisms [54].

The largest endometrial GWAS published to date determined genetic correlations between endometrial cancer and 224 non-cancer traits [19]. A significant positive genetic correlation, consistent with existing epidemiological observations, was found between endometrial cancer risk, type 2 diabetes, and anthropometric traits related to obesity (e.g., body mass index (BMI) and waist circumference) [19]. A significant negative correlation was found between years of schooling and the age of menarche, both of which negatively correlate with obesity-related traits [19].

LD score regression has explored the relationship between endometrial cancer and various cancers, finding a strong correlation with ovarian and ER-positive breast cancer [55,56]. Additionally, LD score regression has unveiled a potential shared genetic architecture with non-cancerous gynecological diseases, including polycystic ovarian syndrome (PCOS), uterine fibroids, and endometriosis [57,58,59]. Unlike the genetic correlation between uterine fibroid and endometrial cancer, adjustment for genetically predicted BMI at least partly mediated the genetic correlation between PCOS and endometrial cancer [59]. Further research into cross-trait genetic correlation will enable a better understanding of endometrial cancer genetic predisposition.

While LD score regression is predominantly used to uncover genetic correlations in relation to endometrial cancer, multiple methods are available, each with their own advantages and limitations. The GeNetic cOVariance Analyzer (GNOVA) is often compared to LDSC for providing fast and accurate estimates, particularly efficient in large datasets [27]. GNOVA employs a method of moments algorithm to estimate genetic covariance, unlike the weighted regression used in LDSC, and has been widely applied to complex phenotypes [60,61,62]. Studies have shown similar results when using both methods to estimate the genetic correlation between sex hormones and breast cancer [63]. However, both LDSC and GNOVA assume a linear relationship between LD scores and test statistics/genetic covariance, which might not hold true for all traits and populations [64].

Phenotype Correlation–Genotype Correlation with summary statistics (PCGC-s) is another tool for genetic correlation. It is designed to correctly model case-control data and outperform LDSC in the presence of covariates representing major risk factors, such as sex and age [23]. PCGC-s has been used in endometrial cancer research to detect positive correlations between ovarian cancer, uterine fibroids, and endometriosis, although the authors stated the approach did not produce standard errors or *p*-values for the estimates [57]. Genomic-SEM uses structural equation modeling to determine an underlying latent factor driving an observed genetic correlation between two traits [21,65]. Though more computationally intensive, it offers greater parameter flexibility to identify the most representative model to fit the data, improving the accuracy of the estimated genetic covariance [21].

When data are available and computational efficiency is not a limiting factor, individual-level data-based methods using restricted maximum likelihood (REML) provide more precise genetic correlation estimates compared to LDSC and other summary-based methods [66,67]. Several individual-level tools have been developed (Table 1), differing primarily in their log-likelihood optimization algorithms [67]. However, data availability often poses a logistical barrier to using individual-level methods, making GWAS summary statistics methods more popular for determining genetic correlations between traits. While a powerful tool for understanding the overall genetic similarity of complex traits, genetic correlation analysis has several limitations to consider. LD score regression employs a polygenic model and is most effective when analyzing traits with a polygenic genetic architecture. However, when significant SNPs account for a sizable proportion of heritability, analyzing only those SNPs can prove more efficient [24]. It is crucial to recognize that genetic correlation analysis cannot establish causal relationships or determine the directionality of effects [24]. Any observed genetic correlation could result from a true direct relationship between two traits, or the genetic variant could be associated with an unknown risk factor, which also affects both traits. Therefore, while significant results may suggest shared genetic architecture, caution is needed in interpreting these findings without additional evidence from functional studies or experimental designs capable of elucidating causal relationships.

The absence of genome-wide genetic correlation does not overrule the occurrence of locus-specific genetic correlation. A correlation between two traits could result from multiple genetic variants, some of which may have opposing effects on the two traits; in these circumstances, local genetic correlations can be more insightful in identifying shared common causes [66]. Regional genetic correlations can quantify which genomic regions disproportionately contribute to the genome-wide correlation [28]. A large-scale cross-cancer study used ρ-HESS (Heritability Estimation using Summary Statistics) to successfully identify thirteen pairs of cancers with statistically significant local genetic correlations across eight distinct genomic regions [68]. This study found a positive genetic correlation between endometrial and prostate cancer at region 17q12 and a statistically significant local genetic correlation at 5p15.33 across six pairs of cancers, including ER-negative breast, pancreatic, glioma, melanoma, lung, pancreatic, prostate, and colorectal cancer. Despite the varying direction of these correlations, the result postulates that the 5p15.33 region may harbor key genetic variants related to multiple cancer types supported by the number of susceptibility variants already reported in this region [68].

Several methods have been developed for estimating local genetic correlation, including ρ-HESS, LAVA (Local Analysis of [co]Variant Annotation), and SUPERGNOVA, each offering unique advantages in elucidating distinct genetic correlations across genomic regions [28,29,30]. While all these methods utilize summary statistics, they differ in their sensitivity to data quality, sample size, and choice of reference panel [69]. ρ-HESS and SUPERGNOVA are primarily focused on bivariate correlation estimates, providing precise measurements for pairwise trait correlations [28,30]. A real-data application comparing the two methods highlighted the importance of reference panel selection in local genetic correlation analyses. The study concluded that SUPERGNOVA is more robust to variations in LD matrices but is susceptible to type-I errors, whereas ρ-HESS maintains well-controlled type-I error rates but sacrifices statistical power [69]. In contrast, LAVA offers a unique capability by extending its application to estimate multivariate correlations, allowing for a more comprehensive analysis of multiple traits simultaneously [29]. This flexibility makes LAVA particularly advantageous in studies utilizing an in-sample reference panel, aiming to uncover complex genetic relationships across various phenotypes.

Pinpointing specific regions that may drive the global genetic correlation, as well as regions that might be neutral or antagonistic, can complement genome-wide analysis and deepen understanding of the genetic architecture of the traits. Such analysis has not been widely pursued in endometrial cancer, thus presenting an area ripe for exploring locally shared genetic pathways that may go unnoticed in genome-wide genetic correlation analysis.

## 4. Colocalization

Colocalization refers to the identification of causal variants shared between different traits after controlling for the independent signals identified in individual analysis [70,71]. Multi-trait colocalization enhances the statistical power to identify shared variants across multiple traits and provides a more robust indication of the variant’s potential causality [31]. Programs developed for colocalization analysis use Bayesian statistical tests to enable the computation of posterior probabilities that can disentangle whether the association signals across traits colocalize (colocalization) or are driven by distinct causal variants (pleiotropy) (Table 1) [31]. While programs such as COLOC [31] look at a specific queried locus, GWAS-PW [32] can perform genome-wide analysis, partitioning the genome into predefined regions.

Colocalization analysis in endometrial cancer research has unveiled intriguing insights into the shared genetic architecture between this malignancy and other traits or diseases. Colocalization analyses have indicated shared genetic variants between endometrial cancer and traits such as ovarian cancer [55] and COVID-19 phenotypes [72]. Colocalization analysis can also include expression quantitative trait loci (eQTLs) that have enabled the identification of several novel candidate endometrial cancer susceptibility genes [59,73,74,75].

Colocalization analysis faces several limitations; for example, in instances of very high linkage disequilibrium (LD), distinguishing between shared pleiotropic variants and those acting independently becomes challenging, leading to ambiguity in attributing shared genetic influences to specific traits. Additionally, limitations in statistical power may hinder the detection of true colocalization events, particularly in independent studies with small sample sizes or low genetic variant density. Like genetic correlation analysis, colocalization analysis does not provide information on the directionality of effects or causality, highlighting the need for complementary experimental approaches to validate findings and elucidate underlying biological mechanisms.

## 5. Cross-Trait Locus Identification

Cross-trait locus identification is the process of identifying genetic variants associated with multiple phenotypes or traits simultaneously. This approach uses a meta-analysis framework to combine summary or individual-level statistics of distinct or related traits to detect pleiotropic loci with shared associations. Cross-trait GWAS leverage power from multiple genetically correlated phenotypes to detect genetic loci that may not reach genome-wide statistical significance when considering each trait individually. Moreover, cross-trait analysis offers the benefit of utilizing existing datasets, obviating the necessity for additional genotyping efforts, thus presenting a considerable practical advantage.

Several methods have been developed for cross-trait variant identification (Table 1), with the most commonly used being Multi-Trait Analysis of GWAS (MTAG) due to its robustness to sample overlap and its ability to leverage GWAS summary statistics from multiple traits simultaneously [33]. MTAG can improve statistical power by borrowing information across traits and has been shown to increase the discovery of associated loci, especially for traits with smaller sample sizes [33]. Additional methods for cross-trait variant identification include Cross-Phenotype Association (CPASSOC), employing a Bayesian framework to account for correlation structures and offering valuable insights into shared genetic mechanisms [34]; Multivariate PLINK (MV-PLINK), which is particularly effective in extensive individual-level datasets, employing multivariate linear regression to detect common variants while controlling for covariates and population structure [35]; and Multi-Phenotype Conditional False Discovery Rate (MultiPhen conjFDR), regulating false discovery rates across diverse traits and highlighting variants with the strongest evidence of shared associations [36,37].

Cross-trait variant identification has shown promise in advancing our understanding of endometrial cancer by uncovering novel risk regions replicable in larger endometrial cancer GWAS. In past studies, joint analyses aimed at identifying cross-trait variants associated with endometrial cancer have employed MTAG, incorporating genetically correlated traits such as ovarian cancer, PCOS, and uterine fibroids [55,59]. Additionally, joint analyses with endometriosis used a fixed-effects GWAS meta-analysis for cross-trait variant identification [58]. Preliminary findings using RE2C [39] supported the identification of five novel risk loci for endometrial cancer in a joint analysis with three hormone-related cancers (breast, prostate, and ovarian) [76]. Other cross-trait analyses of COVID-19 and endometrial cancer employed CPASSOC and determined five independent pleiotropic SNPs for endometrial cancer located at three previously identified loci (9q34.2, 2p16.1, and 15q21.2) [72]. These findings postulate genetic variants can simultaneously affect susceptibility to severe COVID-19 and the risk of developing endometrial cancer through shared biological mechanisms rather than a direct causal link. Severe COVID-19 is characterized by an excessive inflammatory response known as a cytokine storm, which can lead to tissue damage and chronic inflammatory states. This prolonged inflammation may create an environment conducive to cancer development, potentially explaining the observed genetic correlations between severe COVID-19 and endometrial cancer. Similarly, a multi-trait GWAS analysis using a Bayesian framework (bGWAS) [38] included several endometrial cancer risk factors and identified a novel risk locus at 7q22.1, replicated in an independent cohort [74]. This newly identified locus at 7q22.1 encompasses *CYP3A7*, which encodes an enzyme that metabolizes testosterone and synthesizes estrogen. Exposure to estrogen is suspected to elevate the risk of endometrial carcinogenesis. Despite these insightful findings, there is ample room for more research in cross-trait variant identification, including a broader range of traits, to improve our understanding of the underlying genetic architecture of endometrial cancer and shed light on potential biological pathways and mechanisms driving its development.

Strategies aimed at identifying cross-trait loci are diverse and continually evolving. *p*-value combination methods are preferred when only summary statistics are available and computational efficiency is a priority. These approaches represent a significant category of meta-analysis techniques that impose fewer restrictions on statistical modelling, thereby broadening their applicability. Notable examples including TATES, Multi-ACAT, PCSC, and CCT are highlighted in Table 1, where their potential advantages and limitations are discussed [41,42,43,44]. These methods effectively handle diverse and complex data structures, making them a valuable tool in facilitating the integration of multivariate analyses.

Cross-trait variant identification, while demonstrating many potential benefits, also presents several limitations. Firstly, a shared causal variant may exhibit varying biological impacts among different traits, leading to heightened heterogeneity in reported effect sizes. Secondly, a genetic variant might solely influence a subset of study traits. Methods aimed at identifying cross-trait variants typically report an overall association with a genetic locus without attributing the association to a specific trait. Occasionally, the same genetic variant can exert opposing effects, increasing the risk of one disease while decreasing the risk of another. In such instances, these methods may fail to detect an association when a genetic locus exhibits antagonistic effects on the traits under investigation. Lastly, discriminating between heterogeneous effects and statistical noise can prove challenging when assessing multiple traits of differing power and study design. These complexities underscore the need for robust methodologies and cautious interpretation in cross-trait variant identification analyses to ensure reliable insights into shared genetic architecture across diverse traits and diseases.

## 6. Causal Inference Analysis

Mendelian randomization (MR) is the most commonly used genetic approach for causal inference analysis, a statistical method that uses genetic variants as instrumental variables (IVs) to infer causal relationships of observed associations between an exposure trait and an outcome trait [47]. MR is analogous to a conventional randomized controlled trial; however, unlike random intervention allocation, MR relies on the premise that the germline alleles of these variants are randomly distributed (due to Mendel’s Law of Independent Assortment), thereby establishing an unconfounded relationship between exposure and outcome [47,77]. When the exposure traits have a substantial genetic component, the IV explains a larger proportion of variation in the risk factor and improves power. As GWAS samples become larger and more SNP associations are identified, the IVs will only become a stronger proxy for the exposure trait, increasing statistical power in MR studies [66].

**Figure 1:**
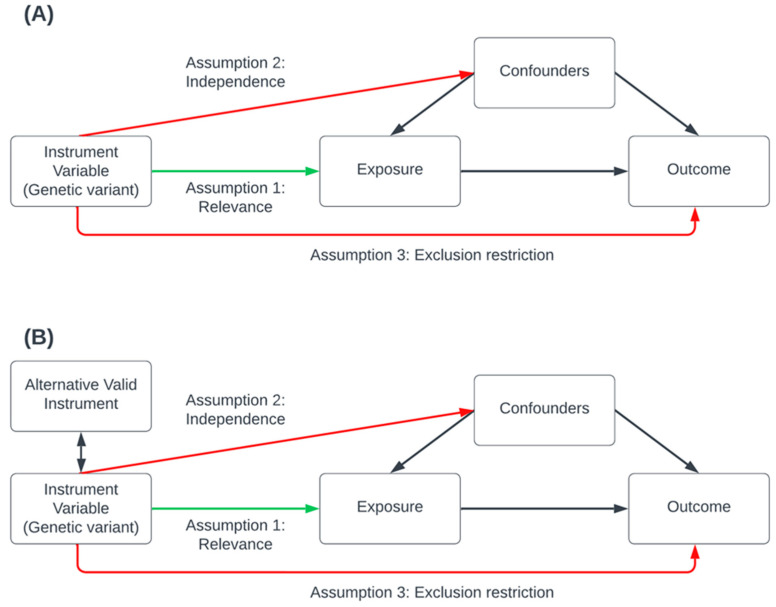
Conceptual visualization of the key genetic instrumental variable assumptions of Mendelian Randomization. In this valid Mendelian randomization (MR) simulation, a genetic variant causally affects the exposure, which may, in turn, causally affect the outcome while accounting for confounders that influence both the exposure and the outcome. Causal effects are depicted using one-sided arrows indicating the direction of causation. Panel (**A**) illustrates the three key assumptions: the green arrow represents the relevance assumption, indicating a valid causal pathway, while the red arrows represent causal pathways explicitly excluded by the independence and exclusion restriction assumptions. Panel (**B**) depicts a scenario in which a valid alternative instrument, though not causal, is in linkage disequilibrium with a causal variant introducing a bias due to pleiotropy. This is tackled by applying a range of MR sensitivity analysis methods and recognition of assumptions underlying the analysis when interpreting results.

Several publications have described the underlying statistical framework and assumptions required for MR [47]. Briefly, for the validity of causal effect estimates, MR analysis relies on three primary assumptions that must be satisfied (Figure 1): the relevance assumption, which asserts that genetic variants are robustly associated with the risk factor; the independence assumption, which stipulates that there are no external confounding influences affecting the association between the genetic variants, the risk-factor, and the outcome; and the exclusion restriction assumption, which posits that the genetic variants affect only the outcome through the risk-factor (also known as the pleiotropy assumption); this assumes there is no pleiotropic effect influencing the gene–outcome association other than that of the vertical pleiotropy implicating the causal pathway between the risk-factor and the outcome. MR methods should be combined with robust estimation methods (Table 2) to tackle bias introduced due to pleiotropy [78].

MR analyses can clarify the causal nature between putative risk factors and endometrial cancer susceptibility [18]. A recent study analyzed all known and suspected risk factors for endometrial cancer risk using MR and then performed a multivariable analysis to distil risk factors into five independent factors: waist circumference (in a module with BMI), age of menarche, age of menopause, SHBG levels, and testosterone levels [74]. Factors such as cigarette smoking, dietary factors, cannabis consumption, gut microbiota, sedentary behavior, and caffeine consumption have all received attention in MR research [92,93,94,95,96,97,98,99]. When interpreting results from published studies, it is crucial to consider the quality of the MR analysis as the quality of evidence provided relies on the satisfaction of instrumental variable assumptions. The accessibility of summary-level data for MR has contributed to a surge in the tool’s popularity, often leading to its opportunistic use without substantiated biological or functional relevance. The risk factor should only be considered a common genetic predictor in these instances. While this represents a weaker claim, it still holds its place in scientific literature. Causation claims in MR analysis should be entirely for genetic variants with well-established biological and statistical relevance. For example, the established relationship between obesity and endometrial cancer risk means obesity-related factors have received extensive focus from MR research. Several studies leveraging GWAS [100,101] data have substantiated the causal link between higher BMI and increased risk of endometrial cancer across multiple European and Japanese cohorts [100,101,102,103,104,105] and histological subtypes [19,100].

MR approaches can unravel the associations between endometrial cancer and a trait that may be affected by their relationships with BMI. For example, childhood adiposity is an apparent health problem epidemiologically associated with endometrial cancer risk [106,107]. However, the causal nature of this association and whether it represents a direct or indirect effect mediated by adult obesity remain unclear. MR studies have disentangled the relationship between child and adult adiposity and subtype-specific endometrial cancer risk, identifying direct independent effects of childhood adiposity on the risk of non-endometrioid endometrial cancer but a minimal indirect effect that adult adiposity mediates on endometrioid EC risk [106]. These novel findings shed light on the critical role of targeting adiposity at different life stages to limit subtype-specific endometrial cancer risk. Similarly, MR analyses have determined molecular mediators underlying endometrial cancer risk [103]. This study identified that two sex-steroid hormones (bioavailable testosterone and SHBG), as well as fasting insulin, strongly mediate the relationship between excess adiposity and endometrial carcinogenesis [103]. These findings suggest that in the future, there is scope to investigate targeting these hormone-related and insulin-related traits for endometrial cancer prevention.

With the rapid advancement of MR in endometrial cancer research, awareness of the limitations of this approach is essential for the correct interpretation of results. As mentioned, traditional approaches rely on the core assumptions being met for accurate causal effect estimates [108]. Bias in MR studies can arise from confounding of genetic-intermediate phenotype–disease associations, trait heterogeneity, and linkage disequilibrium, which may obscure causal effect estimates [89,109,110,111]. Sensitivity analyses and careful selection of instrumental variables are essential to address these issues and ensure reliable causal inferences [111,112]. Additionally, canalization or developmental compensation processes can distort MR estimates by mitigating the effects of genetic variants on phenotype expression, further complicating interpretation [111,112,113]. Other limitations include confounding due to population stratification, dynastic effects, assortative mating, selection bias, and collider bias, all of which can introduce systematic errors and undermine the validity of MR findings [111,114,115].

## 7. Conclusions

In summary, this review underscores the crucial role of cross-trait GWAS in elucidating genotype–phenotype associations and advancing our understanding of complex diseases such as endometrial cancer. It provides an updated synopsis of the genetic architecture of endometrial cancer by comprehensively detailing related genetic studies in the field. Through leveraging large-scale publicly available data, joint analysis has effectively highlighted the interplay between genetic susceptibility in different phenotypes, offering insights into comorbidities, modifiable risk factors, and genetic predisposition. While primary analytic methods have been instrumental in unveiling significant associations with shared common genetic variants in endometrial cancer, these variants only explain a fraction of the expected risk variance, suggesting the potential for introducing new statistical tools to identify novel risk loci. Despite the potential benefits of cross-trait GWAS, they also present methodological challenges, underscoring the need for robust methodologies and cautious interpretation to ensure reliable insights.

Furthermore, this review delineates significant findings achieved through various stages of genome-wide cross-trait analysis, including genetic correlation, colocalization analysis, cross-trait meta-analysis, and Mendelian randomization. It emphasizes that investigation of shared genetic factors in endometrial cancer is still in its infancy, offering numerous promising avenues for future exploration. The expanding data repositories and innovative analytical methodologies enhance the capacity for identifying risk loci. Integrating multi-omics datasets can also deepen our understanding of the molecular mechanisms underlying disease susceptibility and progression, potentially unveiling novel biomarkers. These advancements broaden the scope for downstream analyses focused on discovering new biological pathways and therapeutic targets, with the ultimate goal of clinical translation of multiple diseases. Cross-trait GWAS may facilitate the development of polygenic risk scores and predictive models to assess individual risk profiles and guide personalized prevention and intervention strategies. Moreover, expanding GWAS data to include under-represented populations, thus better reflecting the global community and increasing emphasis on cross-population analyses, may offer novel insights into disease etiology and pathogenesis. The field holds promise for further elucidating the genetic basis of endometrial cancer and other complex diseases, offering multiple avenues for improved prevention, diagnosis, and treatment strategies that can impact on the global community.

## Figures and Tables

**Table 1:** Programs available for exploring relationships between traits using GWAS data.

Program	Ref.	Description	Data Input ^1^	Examples ^2^
Genetic correlation				
BOLT-REML	[20]	A Monte Carlo algorithm for variance component analysis to estimate genetic correlations and partition SNP heritability among multiple phenotypes. Computationally fast.	Individual	37340002 30929738
GenomicSEM	[21]	Synthesizes genetic correlations of multiple traits with unknown amounts of sample overlap.	Summary	32606422 35513722
GCTA	[22]	Provides highly accurate estimates of genetic correlations between phenotypes while accounting for different genetic architectures.	Individual	24944428
PCGC-s	[23]	Estimates genetic correlation and partitioned heritability large datasets while accounting for case-control sampling and covariates.	Summary	31488892
LD Score Regression	[24]	Estimates genetic correlations across multiple phenotypes while accounting for cryptic relatedness and population stratification.	Summary	30093612 29608257 26414676
HDL	[25]	Highly powered and accurate estimates of genetic correlations fully account for whole genome LD and reduce the variance of genetic correlation estimates.	Summary	35492879 34997191
MTG2	[26]	Combines the average information algorithm used by REML with an eigen-decomposition of the genomic relationship matrix to estimate genetic variance.	Individual	29977057 35729236
GNOVA	[27]	Provides powerful statistical inference through annotation-stratified genetic covariance analysis that is robust to LD and sample overlap.	Summary	37034223 34634379
ρ-HESS	[28]	Localized and precise quantification of genetic correlation between pairs of traits due to small-region genetic variation. Accounts for LD and sample overlap while making no distributional assumptions on the causal effect size under a fixed effects model.	Summary	34355204 34561436
LAVA	[29]	Tests local genetic correlation between two phenotypes. Can also analyze local heritability and conditional genetic relationships between several phenotypes.	Summary	36471075 38637617
SUPERGNOVA	[30]	Accurate and powerful local genetic correlation estimate using summary statistics that is robust to arbitrary and unknown amount of sample overlap.	Summary	38191017 37467357
Colocalization				
COLOC	[31]	Bayesian statistical test to enable the computation of probabilities that two traits share a common genetic causal variant from single variant association *p*-values and MAFs. Locus-specific analysis.	Summary	34268601 34355204
GWAS-PW	[32]	Bayesian statistical test to enable the computation of probabilities that two traits share a common genetic causal variant from single variant association *p*-values and MAFs. Locus-specific analysis.	Summary	33144283 35178771 35851147
Cross-Trait Locus Identification				
MTAG	[33]	Joint analysis of multiple traits to increase statistical power and account for sample overlap.	Summary	34268601 31488892 29292387
CPASSOC	[34]	Assess cross-phenotype associations for both continuous or binary traits.	Summary	37636041 31669095
MV-PLINK	[35]	Computationally fast implementation of canonical correlation analysis, including multiple phenotypes and uses	Individual	35278618
MultiPhen	[36]	Employs ordinal regression for joint multivariate modelling of multiple phenotypes, with increased statistical power and an appropriate type 1 error rate.	Individual	35701404 35680855
conjFDR	[37]	A model-free strategy for analysis that leverages genetic overlap between two phenotypes which boosts statistical power and identifies shared genomic association regardless of the cross-trait correlations.	Summary	37752828 31792363
bGWAS	[38]	A Bayesian method that leverages published studies for related risk factors to construct priors. Increase power to identify susceptibility variants and allows for assessment of posterior and direct effects.	Summary	37168552 35653391
RE2C	[39]	A generalized likelihood model that accounts for correlations of statistics and achieves optimal power under the condition of heterogeneity.	Summary	35492870 3734002 35753705
MetABF	[40]	Employs a Bayesian framework using both an independent and fixed effect model to meta-analysis GWAS statistics. An efficient tool that allows the expected relationships between studies or traits to be encoded in the analysis.	Summary	36653479 35492870
TATES	[41]	A multivariate method that combines univariate GWAS *p*-values to estimate a global trait-based *p*-value while accounting for correlations between phenotypes. Increase power to identify novel susceptibility variants.	Summary	35391794
Multi-ACAT	[42]	Computationally fast and flexible combination *p*-value method to test for association with a single rare or common variants and multiple phenotypes in a genomic region	Summary	33432394
PCSC-s	[43]	Cauchy combination method to test the association multiple phenotypes and a variant using an integrated *p*-value. Particularly effective in joint-analysis of phenotypesfrom unbalanced case-control association studies.	Summary	36691904
CCT	[44]	Combination *p*-value method in which test statistic is a weighted sum of Cauchy transformation of individual *p*-values. Powerful under arbitrary dependency structures of the *p*-values but lacks power when large and small *p*-values are combined.	Summary	35210502
Causal Inference				
LCVA	[45]	Distinguishes causal relationships among genetically correlated phenotypes such that a positive result is more likely to be the true causal effect.	Summary	36653534 36151087 31669095
MiXer	[46]	Applies a bivariate causal model to quantify and visualize polygenic overlap by estimating the total number of shared and trait-specific causal variants.	Summary	37752828 34761251
Mendelian randomization	[47]	Uses instrument variables in statistical models to identify causal relationships between an exposure and outcome. Various programs and techniques have been developed (see Table in Section 6).	Both	34268601

^1^ GWAS data required for analysis: Individual-level genotypes, or summary statistics, ^2^ Example publications (PubMed IDs) that have used these approaches; note, this is not exhaustive.

**Table 2:** Mendelian randomization software packages and consistency assumption approaches.

Methods	Ref.	Description
Packages		
MR-Base	[79]	A web platform housing GWAS summary statistics that can perform two-sample MR analyses.
MendelianRandomization	[80]	R software package that implements several methods for MR analyses based on summary statistics including multivariable MR.
CAUSE	[81]	R software package for MR analysis accounting for both uncorrelated and correlated horizontal pleiotropy effects.
TwoSampleMR	[82]	R software package to perform a range of two-sample MR analyses using GWAS summary data from two independent exposure and outcome cohorts.
OneSampleMR	[83]	R software package to perform a range of one-sample MR analyses using GWAS data from a single cohort (individual-level data).
Consistency Assumption: Instrument Strength Independent of Direct Effect		
MR-Egger	[84]	A sensitivity analysis tool used to test for bias from pleiotropy caused by some genetic variants having multiple proximal phenotypic correlations, making them invalid instrumental variables. Egger’s test provides a valid causal effect estimate when some or all the genetic variants are invalid instrumental variables.
Consistency Assumption: Majority Valid		
Weight-median	[85]	A sensitivity analysis tool that uses GWAS summary data for MR with multiple genetic variants. Provides a consistent causal effect estimate even when up to 50% of the information comes from invalid instrumental variables.
Consistency Assumption: Plurality Valid		
Weighted-MBE	[86]	A sensitivity analysis tool using summary data that is robust to horizontal pleiotropy. Provides a consistent causal estimate when the largest number of similar individual-instrument causal effect estimates comes from valid instruments, even if the majority of instruments are invalid.
Consistency Assumption: Horizontal pleiotropy around 0		
MR-LDP	[87]	An efficient variational Bayesian expectation-maximization algorithm using GWAS summary statistics to estimate the causal effects of complex traits that have multiple instrumental variants within LD. The random component eliminates the impact of horizontal pleiotropy.
MR-RAPS	[88]	Uses GWAS summary data under a random-effect model to estimate the causal effects of genetic variants while accounting [81] for pleiotropy. It is robust to outliers but sensitive to the assumption that pleiotropy is normally distributed around 0.
Consistency Assumption: Outlier-robust		
GSMR + HEIDI	[89]	Uses summary GWAS data to perform MR analysis by accounting for LD between the variants, thereby improving statistical power. Detects and accounts for outliers that could violate MR assumptions.
MR-GRAPPLE	[90]	Uses GWAS summary statistics to identify multiple pleiotropic pathways and determine the causal effect, under a likelihood model pervasive pleiotropy accounted for as long as the InSIDE assumption holds for all genetic instruments.
MR-Lasso	[78]	Extension of the IVW-MR framework by adding an intercept term for each genetic variant and a lasso penalty term for identification. Aims to remove a potential source of bias (outliers) that could violate the assumptions of the analysis.
MR-Robust	[78]	IVW method is performed by regression resulting in MM-estimation (robust against influential points) and Tukey’s loss function (robust against outliers). Aims to downweigh outliers which could cause a violation of the assumptions underlying the analysis.
MR-PRESSO	[91]	Uses summary-level data to test and correct for horizontal pleiotropic outliers. Uses aregression framework with a “leave-one-out” approach to detect and remove outliers from the analysis determining which SNP is driving the difference in computed residual sum of squares.

## Data Availability

No new data were created or analyzed in this study. Data sharing is not applicable to this article.
